# Cutinsomes and lipotubuloids appear to participate in cuticle formation in *Ornithogalum umbellatum* ovary epidermis: EM–immunogold research

**DOI:** 10.1007/s00709-014-0623-2

**Published:** 2014-03-14

**Authors:** Maria Kwiatkowska, Agnieszka Wojtczak, Katarzyna Popłońska, Justyna Teresa Polit, Dariusz Stępiński, Eva Domίnguez, Antonio Heredia

**Affiliations:** 1 Department of Cytophysiology, Faculty of Biology and Environmental Protection, University of Łódź, Pomorska 141/143, 90-236 Łódź, Poland; 2 Instituto de Hortofruticultura Subtropical y Mediterránea “La Mayora” UMA-CSIC, Universidad de Málaga, Campus de Teatinos, 29071 Málaga, Spain

**Keywords:** Cuticle, Cutinsomes, Electron microscopy–immunogold technique, Lipotubuloids, *Ornithogalum umbellatum* ovary epidermis

## Abstract

The outer wall of *Ornithogalum umbellatum* ovary and the fruit epidermis are covered with a thick cuticle and contain lipotubuloids incorporating ^3^H-palmitic acid. This was earlier evidenced by selective autoradiographic labelling of lipotubuloids. After post-incubation in a non-radioactive medium, some marked particles insoluble in organic solvents (similar to cutin matrix) moved to the cuticular layer. Hence, it was hypothesised that lipotubuloids participated in cuticle synthesis. It was previously suggested that cutinsomes, nanoparticles containing polyhydroxy fatty acids, formed the cuticle. Thus, identification of the cutinsomes in *O. umbellatum* ovary epidermal cells, including lipotubuloids, was undertaken in order to verify the idea of lipotubuloid participation in cuticle synthesis in this species. Electron microscopy and immunogold method with the antibodies recognizing cutinsomes were used to identify these structures. They were mostly found in the outer cell wall, the cuticular layer and the cuticle proper. A lower but still significant degree of labelling was also observed in lipotubuloids, cytoplasm and near plasmalemma of epidermal cells. It seems that cutinsomes are formed in lipotubuloids and then they leave them and move towards the cuticle in epidermal cells of *O. umbellatum* ovary. Thus, we suggest that (1) cutinsomes could take part in the synthesis of cuticle components also in plant species other than tomato, (2) the lipotubuloids are the cytoplasmic domains connected with cuticle formation and (3) this process proceeds via cutinsomes.

## Introduction

A cuticle, a structure that covers aerial surfaces of terrestrial plants, has various functions: prevention of non-stomatal water loss, inhibition of organ fusion during development (Sieber et al. 2000; Heredia 2003), protection from UV radiation damage (Barnes et al. 1996) and imposition of a physical barrier against infection by bacterial and fungal pathogens (Jenks et al. 1994). Plant cuticles are characterized by their heterogeneous chemical nature. Biopolyester cutin, an insoluble hydrophobic matrix of polyhydroxylated C16 and/or C18 fatty acids cross-linked by ester bonds, is the main component of a cuticle (Pollard et al. 2008). A fraction of waxes is deposited on the surface (epicuticular waxes) and embedded in the cutin matrix (intracuticular waxes). Cutan is another lipid polymer sometimes present in plant cuticles, either as an alternative to or in combination with cutin (Villena et al. 1999; Kolattukudy 2001).

Cuticle components are synthesised in epidermal cells. This process can be divided into two stages: (1) chemical transformation of fatty acids synthesised in plastids into cutin and wax building blocks and (2) polymerisation and transport of the aforementioned oligomers to the epidermal surface. The first stage is mediated by numerous genetically controlled enzymes (Pollard et al. 2008).

During the past decade, significant progress was made in understanding cutin synthesis in *Arabidopsis thaliana* (Bonaventure et al. 2004; Franke et al. 2005; Molina et al. 2006). Genetic and biochemical studies have led to the identification of several transcription factors (Matas et al. 2011; Seo et al. 2011; Wu et al. 2011), genes and proteins required for the synthesis of cutin (Benveniste et al. 1998; Wellesen et al. 2001; Schnurr et al. 2004; Xiao et al. 2004; Bessire et al. 2007; Molina et al. 2008; Li-Beisson et al. 2009; Lü et al. 2009; Weng et al. 2010; Yang et al. 2010; Li et al. 2012; Pulsifer et al. 2012).

One of the greatest challenges of cuticle research is to understand how a hydrophobic polymer or its polyhydroxylated fatty acid precursors can be efficiently transported across the hydrophilic cytoplasm and cell wall to the epidermal surface. It was postulated that the transport of cuticle precursors through the plasma membrane could involve ABC transporters, and recently, WBC11, WBC12 and ABCG transporters have been found to be important for both wax and cutin accumulation on the epidermal surface (Bird et al. 2007; Luo et al. 2007; Panikashvili et al. 2007; Ukitsu et al. 2007; Bird 2008; Kuromori et al. 2010; Chen et al. 2011). Lipid transfer proteins (LTPs) were also postulated to be required for lipid export to the plant surface (DeBono et al. 2009; Yeats et al. 2010; Wang et al. 2012) by vesicular or non-vesicular transport (Lev 2010; Prinz 2010; Samuels and McFarlane 2012).

On the other hand, a chemical method for cuticle synthesis was elaborated on the basis of extensive biotechnological research on biopolyester in vitro synthesis (Benίtez et al. 2004; Heredia-Guerrero et al. 2008, 2011; Domίnguez et al. 2010). They showed that cutin monomers had bifunctional chemical groups with the potential to bind; according to polymer science, this indicates that they are able to generate a nonlinear, amorphous and cross-linked polymer (Domίnguez et al. 2011). Moreover, the location of primary and secondary hydroxyl groups in cutin monomers confers self-assembly properties on them. Cutin monomers, with the right orientation and at the given molecular density, are able to generate structures based on short-range interactions such as hydrogen bonds and other weak interactions followed by chemical polymerisation. Due to these self-assembly and self-polymerisation properties of cutin monomers under specific chemical conditions, polyhydroxy fatty acid nanoparticles, designated cutinsomes, become self-assembled (Heredia-Guerrero et al. 2008, 2009). Cutinsomes are structures of about 50–200 nm in diameter which have liquid-like content and a non-rigid esterified phase with hydroxyl groups in a hydrogen-bonded network. The carboxylate/carboxylic (−COO/–COOH) phase defines the nanoparticle hydrophilic shell separating the hydrophobic lipidic content from the aqueous medium (Heredia-Guerrero et al. 2011). With the use of electron microscopy (EM)–immunogold method with antibodies raised against cutinsomes, their presence was demonstrated *in planta* at the early stages of tomato fruit cuticle development 5 days after anthesis (Domίnguez et al. 2010). Hence, the accumulation and fusion of cutinsomes at the outer side of the epidermal cell wall was proposed as the mechanism for early cuticle formation.

Recently, strong support for extracellular conception of cuticle formation mediated by acyltransferase CD1 (cutin synthase) has been presented in the literature (Yeats et al. 2012). Studies of the transcript level of CD1 showed that this enzyme was synthesized intensively only at the stage of dynamic growth, whereas earlier, only traces of cutin synthase were observed (Yeats et al. 2012). Surprisingly, the authors revealed a small amount of cutin (10–15 % in relation to the wild type) in the pericarp epidermis of the tomato cutin-deficient 1 (*cd1*) mutant which does not have cutin synthase. On the basis of the available information, we hypothesise that the results of Heredia-Guerrero et al. (2008, 2011), showing that only early cutin (procuticle) is organized by the aggregation and fusion of cutinsomes which are spontaneously formed in the epidermis from fatty hydroxyl acids without enzyme involvement, are not contradictory to those of Yeats et al. (2012), but supplementary to them. Then, when a procuticle is being created by cutinsomes, molecules of cutin synthase may be anchored in it and further cuticle synthesis is mediated by cutin synthase. The possibility of non-enzymatic mechanisms of cutin synthesis (as an alternative way to enzymatic synthesis) was also proposed by Yeats and Rose (2013). According to Pollard et al. (2008), the course of cutin synthesis seems to depend on the timing of organ, tissue and species development as well as on polymer localization and function. Hence, it is not surprising that the establishment of general rules regarding the process of cuticle development is a very difficult and complex problem which is still not properly addressed.

Lipotubuloids of *Ornithogalum umbellatum* ovary epidermis are cytoplasmic domains with agglomerations of lipid bodies connected with microtubules, containing microfilaments, endoplasmic reticulum (ER), ribosomes, and a small number of mitochondria, dictyosomes, microbodies and autolytic vacuoles. They do not have their own membrane, but are surrounded by a tonoplast and exhibit progressive-rotary motion as one body (Kwiatkowska 1973; Kwiatkowska et al. 2005, 2009, 2012a).

Lipotubuloids synthesise lipids since they incorporate ^3^H-palmitic acid (20–25 μCi/ml and specific activity of 50 mCi/mmol; Kwiatkowska 1972a, 2004). The labelling of lipotubuloids disappears after extraction with organic solvents, which proves that radioactive molecules are incorporated into lipids. After 6-h post-incubation in a non-radioactive medium, some molecules marked with autoradiographic silver grains labelled the cuticular layer and, similarly to the cutin matrix, were insoluble in an organic solvent. However, some grains leaving lipotubuloids during the non-radioactive post-incubation disappeared after lipid extraction. They might be connected with waxes, soluble in the organic solvent. Hence, we focused our attention on the cuticle (Kwiatkowska et al. 2012b).

The aim of the current paper was to verify the following hypotheses: (1) cutinsomes are involved in cuticle formation and (2) cutin synthesis mediated by cutinsomes takes place in lipotubuloids.

## Materials and methods

### Material

Epidermis of *O. umbellatum* ovary from the sixth to eighth stages of its development (Kwiatkowska et al. 2007) was used. The epidermal cells were non-dividing in the phase of intense elongation. Moreover, parenchymal cells located just under the epidermis (subepidermal parenchymal cells) were used as the control in order to compare their labelling with epidermal cells after the use of immunogold technique with the cutinsome antibody.

### Cutinsome antibody generation

The whole procedure concerning antibody production was carried out according to Domίnguez et al. (2010). In short, cutinsomes were generated in vitro in 50 mM aqueous solution of 9(10),16-dihydroxyhexadecanoic acid at pH decreasing from 12.5 to 6.8 (Apel et al. 2002). Immunized animal responses to the immunogene were assessed with ELISA tests. Successful generation of antibodies that recognize epitopes related to the molecular arrangement and the surface structure of cutinsomes is clear evidence of the chemical identity and stability of these nanoparticles (Domίnguez et al. 2010).

### Immunogold electron microscopy

The samples were fixed in a mixture of freshly prepared 2.5 % glutaraldehyde and 1 % OsO_4_ (1:1) in cacodylate buffer (pH 7.4) for 1 h and post-fixed in 1 % OsO_4_ in the same buffer at 4 °C for 1 h. After dehydration in an ethanol series, the samples were embedded in Epon 812. Ultrathin sections were cut with a Reichert Jung Ultracut ultra-microtome with a glass knife and mounted on nickel-Formvar-coated grids for immunogold–EM study. Prior to the immunogold reaction, the sections were treated with 10 % hydrogen peroxide for 15 min to remove osmium, which changes the antigen structure (Bendayan and Zollinger 1983), and washed in distilled water and finally in PBS (0.01 M, pH 7.4, Sigma). Air-dried grids with the sections were blocked with 0.5 % BSA and 0.05 % Tween-20 in PBS for 20 min, dried with tissue paper and incubated with the primary mice polyclonal anti-cutinsome antibody in the antibody diluent (Dako) 1:25 (overnight at 20 °C). The grids were then washed ten times for 5 min each in PBS and incubated with the secondary antibody conjugated with 20 nm colloidal gold (no. EM GAR20) diluted 1:50 in the antibody diluent for 1.5 h at the same temperature and rinsed again in PBS and in distilled water (ten times for 5 min each). Material not treated with the primary antibody was the negative control. Whole ultrathin sections were double-stained with uranyl acetate and lead citrate (Reynolds 1963) and examined and photographed in a JEOL JEM 1010 transmission electron microscope at 80 kV acceleration voltage.

### Statistics

Two ovaries from each of three plants were taken for immunogold studies. Five images from each ovary which gives 30 cells in total (*N* = 30) were analysed.

The density of labelling, i.e. the number of gold grains per area unit (1 μm^2^), of particular subcellular compartments in epidermal and parenchymal cells of *O. umbellatum* ovary was estimated after the use of the antibody against cutinsomes. Thirty micrographs were analysed for each cell type. Mean values of labelling densities and standard deviations (±SD) were calculated by means of Microsoft Excel spreadsheet.

Statistical significance of differences between values representing labelling densities of particular subcellular compartments in a given cell type were estimated using Student’s *t* test (*p* < 0.05) and Statistica 8.0 Inc. (USA) Computer software.

## Results

During the growth of an ovary, epidermal cells do not divide but enlarge approx. 30-fold; therefore, cutin is synthesised throughout the whole development of an ovary and its transformation into fruit (Kwiatkowska et al. 2007).

**Fig. 1:**
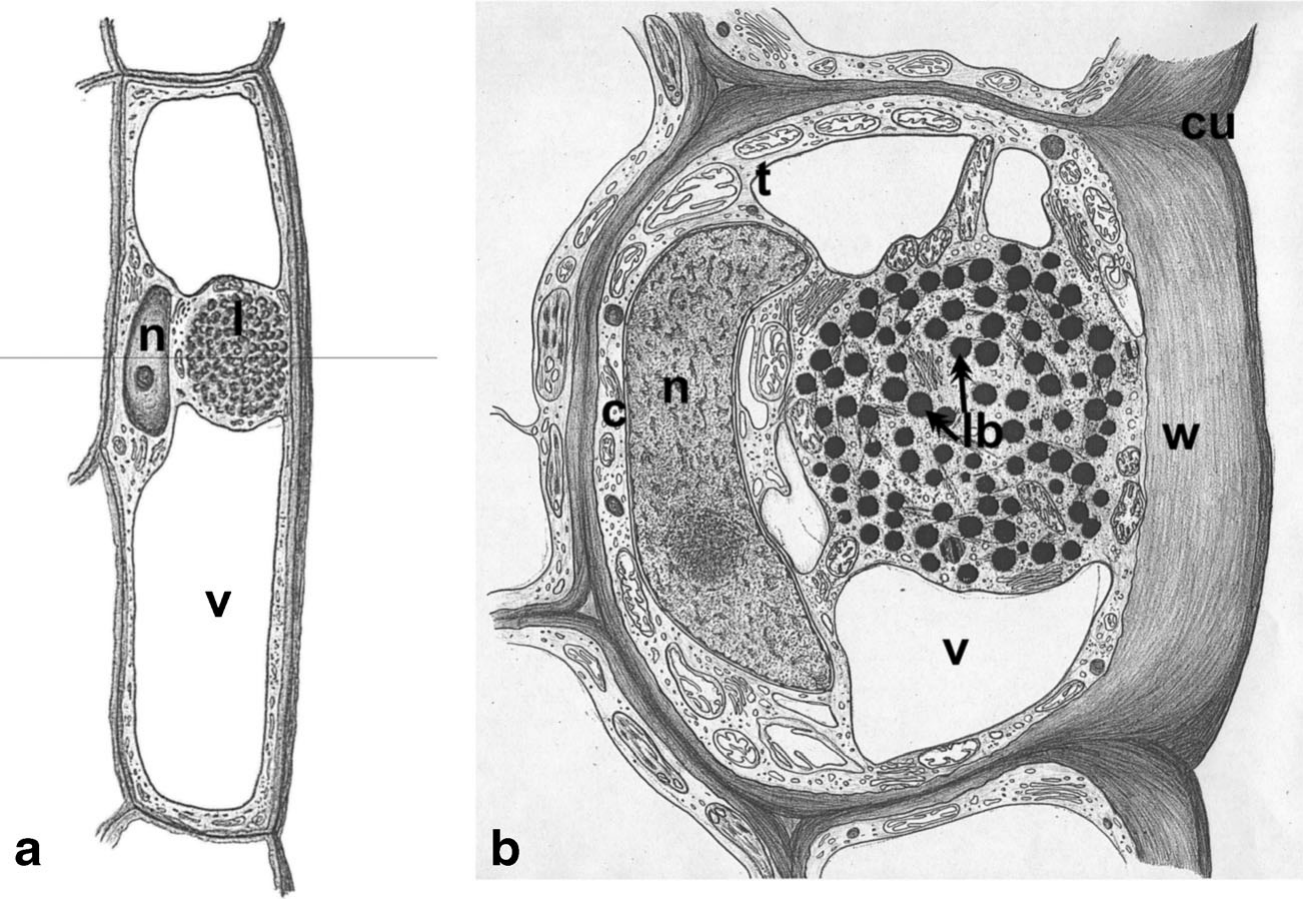
Scheme of *O. umbellatum* ovary epidermal cell from blooming flowers. **a** Longitudinal section of the cell; *vertical line* indicates the site of cross-section. **b** Cross-section of the cell at the site of lipotubuloid placement. *c* cytoplasm, *cu* cuticle, *l* lipotubuloid, *lb* lipid bodies, *n* nucleus, *t* tonoplast, *v* vacuole, *w* polysaccharide wall

Our previous studies were mainly carried out on longitudinal epidermis sections allowing the in vivo observation of lipotubuloids. In the present studies, cross-sections were necessary to visualize the relation between lipotubuloids and cuticle. The experiments were conducted on intensively growing ovary epidermis of blooming flowers. Figure 1 shows the mutual spatial relation between lipotubuloids, outer epidermal wall and cuticle in longitudinal (Fig. 1a) and cross (Fig. 1b) sections. A lipotubuloid dynamically moving and rotating around its changing axis in a cell was caught during fixation and is visible on the ultrathin cross-section near the outer epidermal wall to which it was connected via the cytoplasm.

**Fig. 2:**
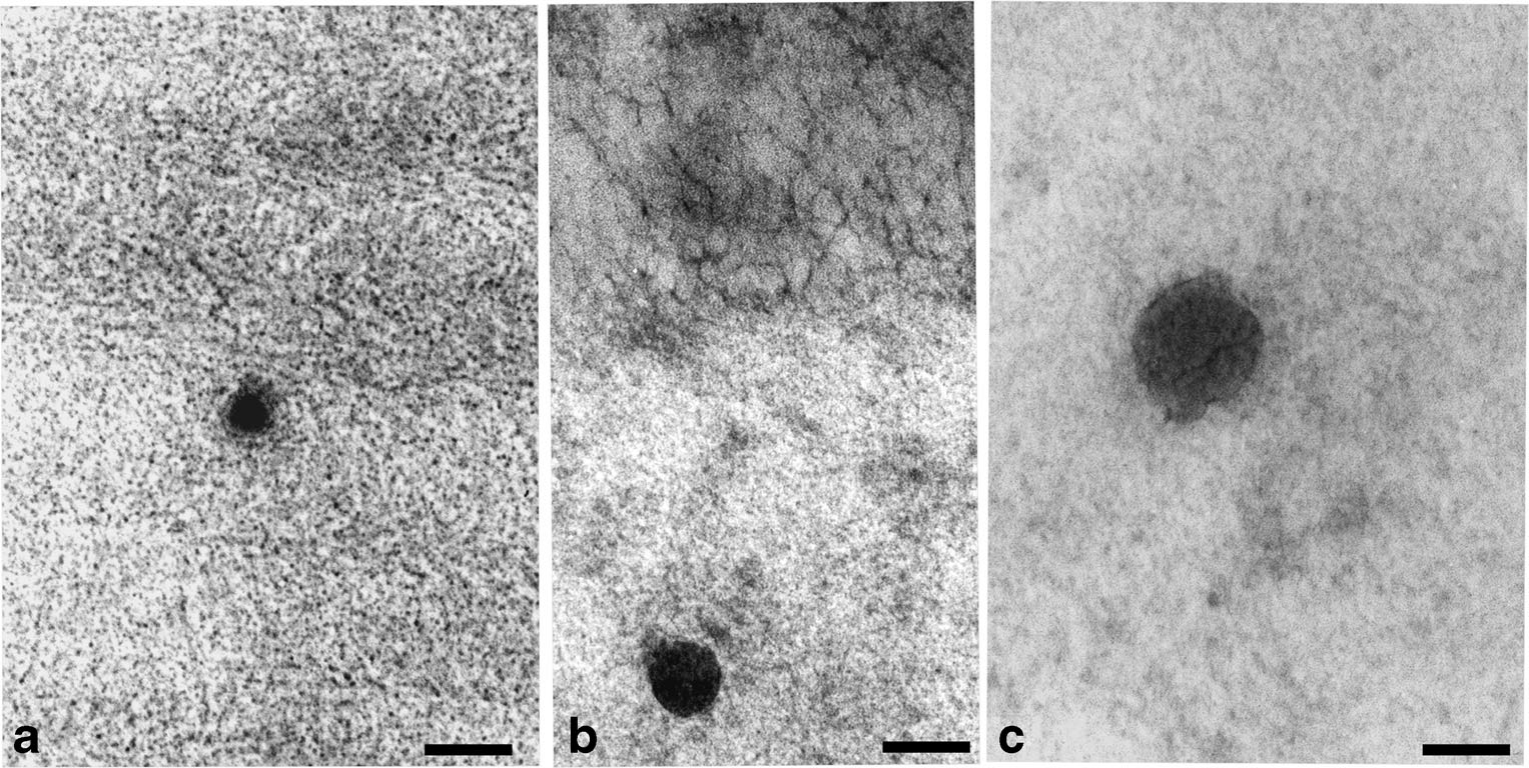
**a**–**c** Cutinsome-like structures in the cell wall of *O. umbellatum* ovary epidermal cells identified by conventional electron microscopy (sections not treated with H_2_O_2_). *Scale bar*, 50 nm

EM morphological observations at a large magnification (×50,000 original magnification) of lipotubuloid revealed dark structures resembling cutinsomes, i.e. in vitro-derived polyhydroxy fatty acid nanoparticles, which were demonstrated by Heredia-Guerrero et al. (2011) using electron microscopy techniques. These cutinsomes were osmiophilic structures of 50–200 nm in size. They assumed various shapes, sometimes irregular, with more or less pronounced dark contour (see Fig. 2 in Heredia-Guerrero et al. 2011). In *O. umbellatum* aerial epidermal cells, cutinsome-like structures were of similar sizes and were located in lipotubuloids, external cell wall (Fig. 2a–c), cytoplasm, near lipid bodies, ER, microtubules and plasmalemma. They were also observed in the cuticular layer and in the area of cuticle proper. Due to the lack of precise morphological criteria for cutinsomes, cutinsome-like structures might be regarded as artefacts. For this reason, it was necessary to undertake their identification using the immunogold method with the use of antibodies against cutinsomes.

**Fig. 3:**
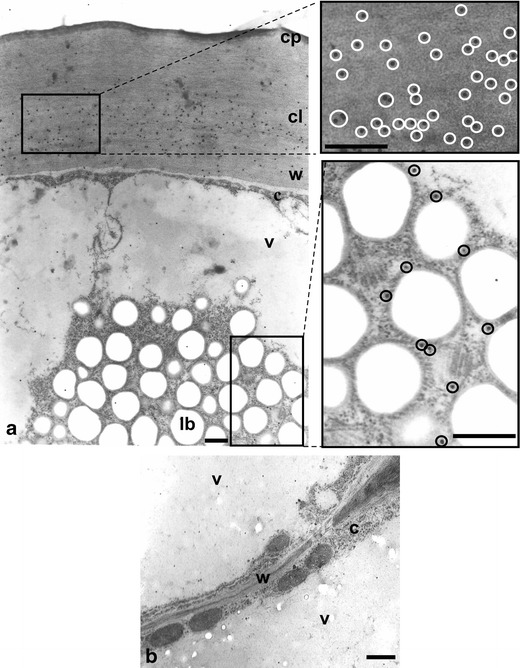
Cross-sections of *O. umbellatum* ovary epidermis and parenchyma cells after immunogold reaction with anti-cutinsome antibody. **a** Part of an epidermal cell with lipotubuloid, the cuticular layer and lipotubuloid labelled with gold particles. Fragments of images in windows are shown to the *right* in higher magnification. Gold particles are highlighted with *circles*. **b** Fragment of parenchyma cells with cell wall lacking gold particles. *c* cytoplasm, *cl* cuticular layer, *cp* cuticle proper, *lb* lipid bodies, *v* vacuole, *w* cell wall. *Scale bars*, 0.5 μm (**a**), 1 μm (**b**)

It is known that all elements involved in cuticle synthesis are localized in those epidermal cells which are covered with cuticle. We compared the numbers of gold grains which labelled cutinsomes in ovary epidermis containing lipotubuloids with those in subepidermal parenchymal cells without lipotubuloids (Fig. 3 and Table 1). Abundant labelling was observed at the territory of an epidermal cell, especially at the area of its outer wall, and of lipotubuloids (Fig. 3a), whereas all parenchyma cells were scarcely labelled (background; Fig. 3b and Table 1). Of the whole labeling, 76 % was observed in the cell wall with cuticle, 15 % in lipotubuloids and the remaining 9 % located in the other components (Table 1). Both types of cells in which the antibody to cutinsomes was omitted were not labelled (negative control, not shown).

**Table 1:** Labelling densities (number of gold particles per 1 μm^2^) of cellular compartments of *O. umbellatum* ovary epidermal and parenchyma cells

Cellular compartment	Cell type	
	Epidermal cells	Parenchyma cells
Cell wall	3.2 ± 0.430*	0.2 ± 0.030*
Cytoplasm and vacuole	0.4 ± 0.038	0.3 ± 0.041
Lipotubuloid	0.6 ± 0.072	–

*p < 0.05

**Fig. 4:**
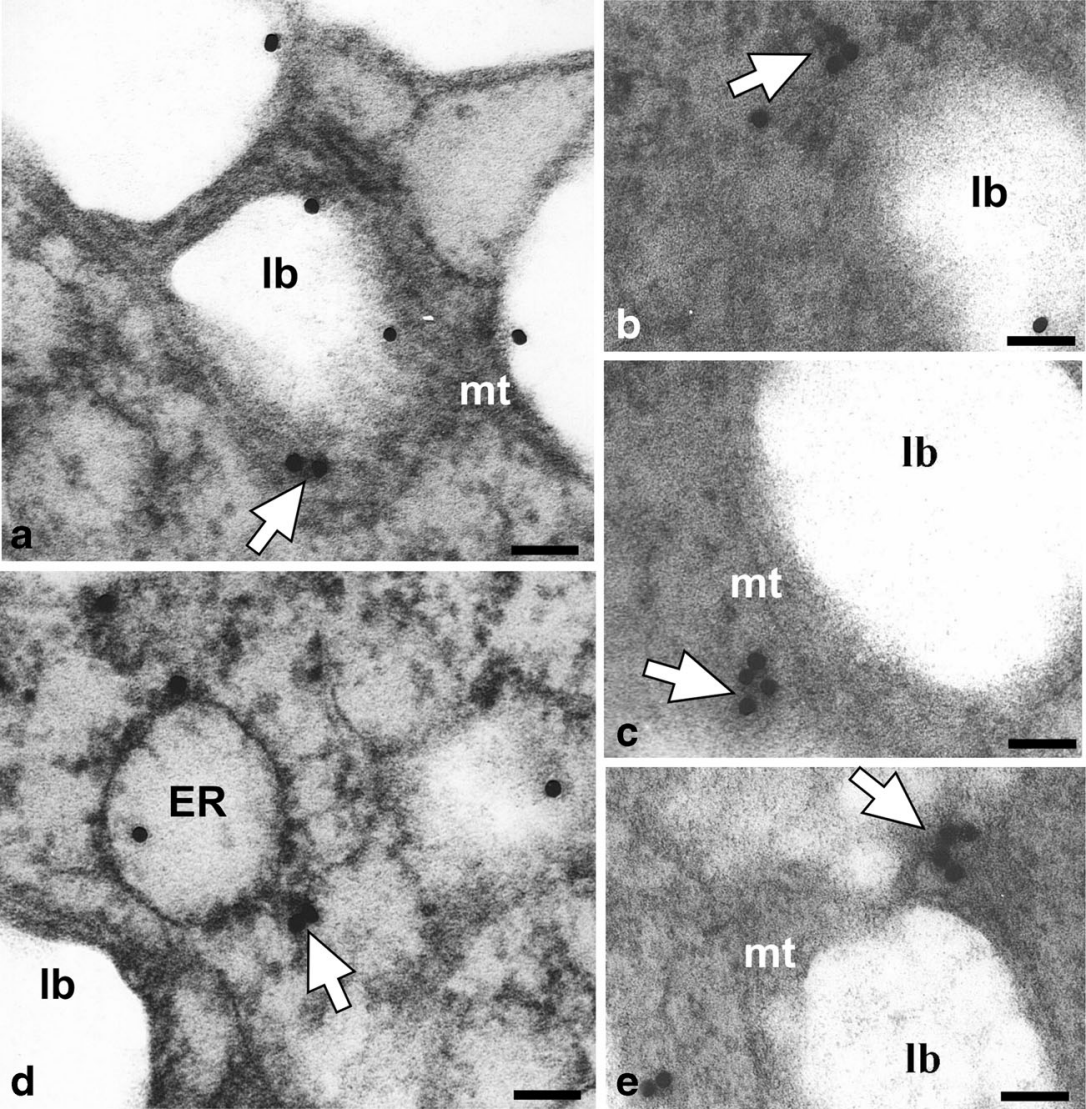
**a**–**e** Cutinsomes identified with immunogold technique in *O. umbellatum* ovary epidermis lipotubuloids (*white arrows*), sections treated with H_2_O_2_. *ER* rough endoplasmic reticulum, *lb* lipid bodies, *mt* microtubules. *Scale bar*, 100 nm

**Fig. 5:**
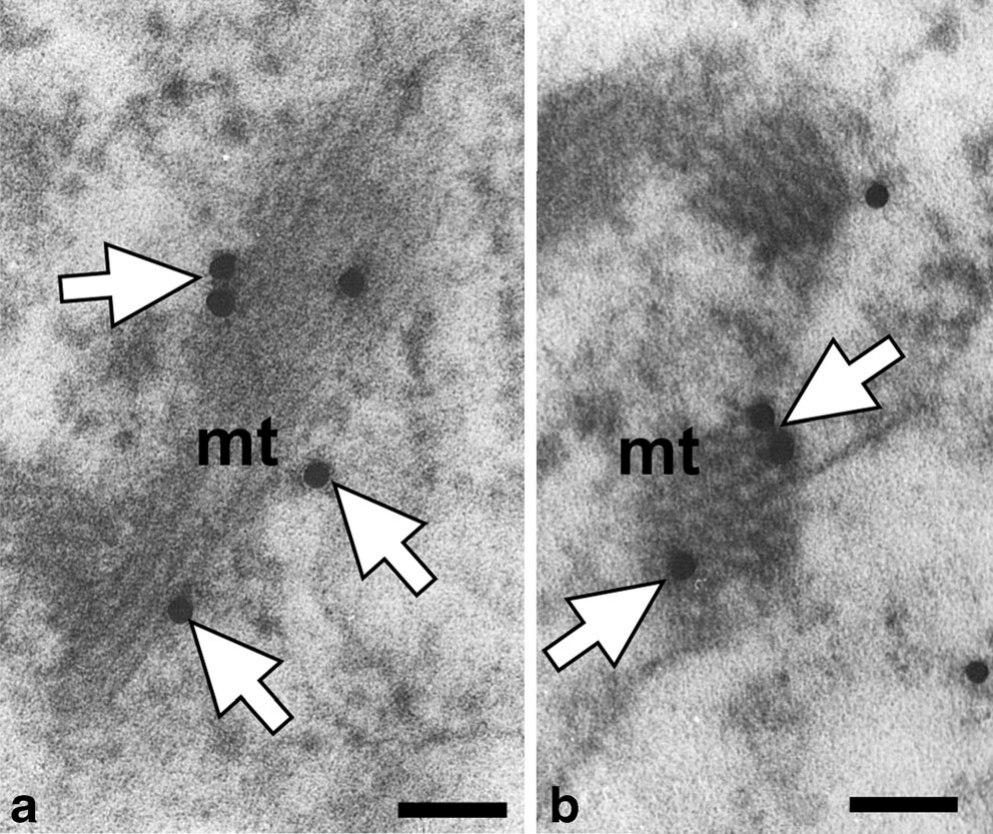
Cutinsomes *in statu nascendi* (*white arrows*) near the microtubule band (*mt*) on longitudinal (**a**) and cross (**b**) sections. *Scale bar*, 100 nm

**Fig. 6:**
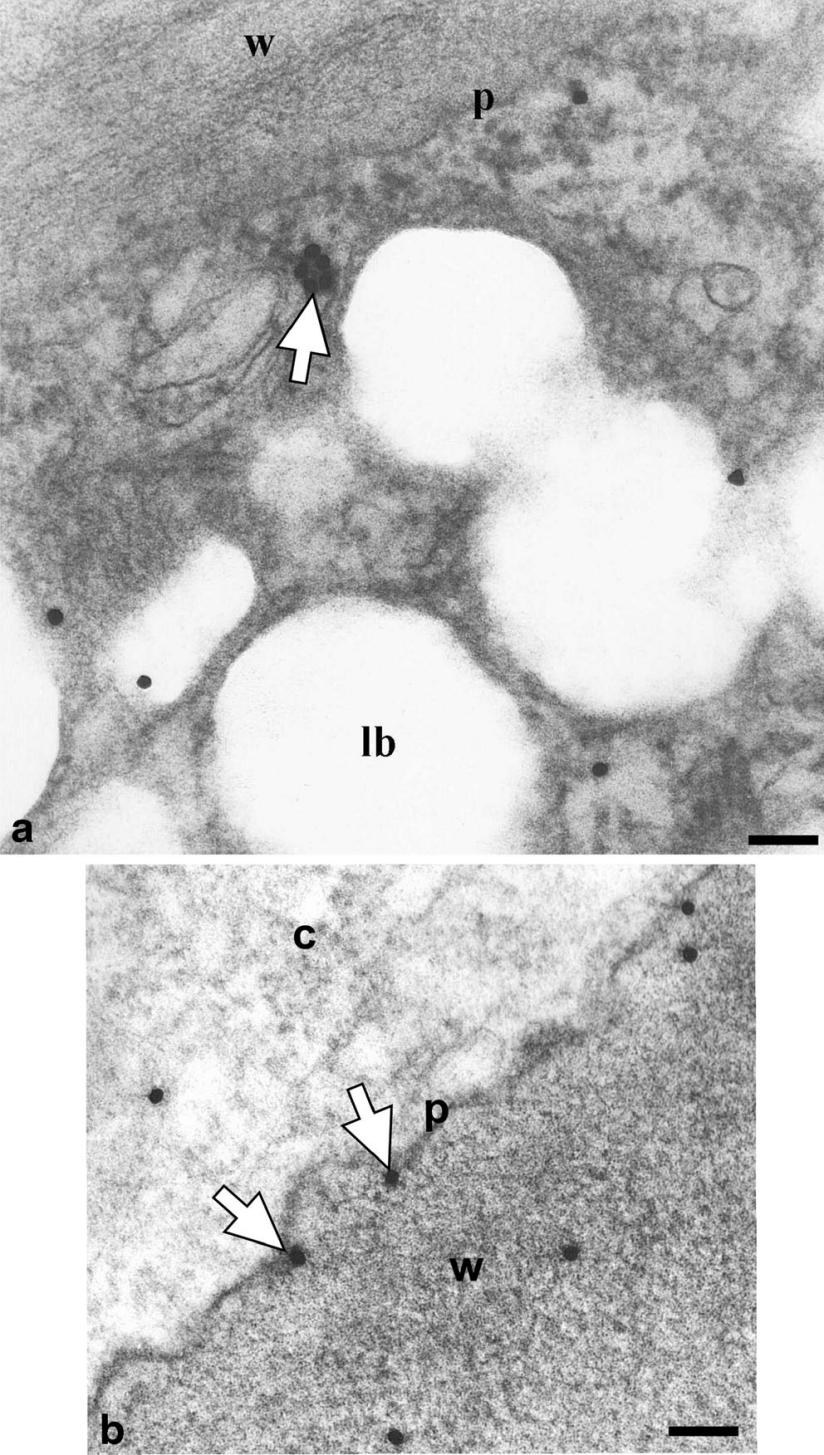
EM immunogold identification of cutinsomes in *O. umbellatum* ovary epidermal cell. **a** Labelled cutinsome (*white arrows*) probably leaving the lipotubuloid. **b** Cutinsomes (*white arrows*) out of the cytoplasm at the other side of the plasmalemma and in the cell wall. *c* cytoplasm, *lb* lipid bodies, *p* plasmalemma, *w* cell wall. *Scale bar*, 100 nm

**Fig. 7:**
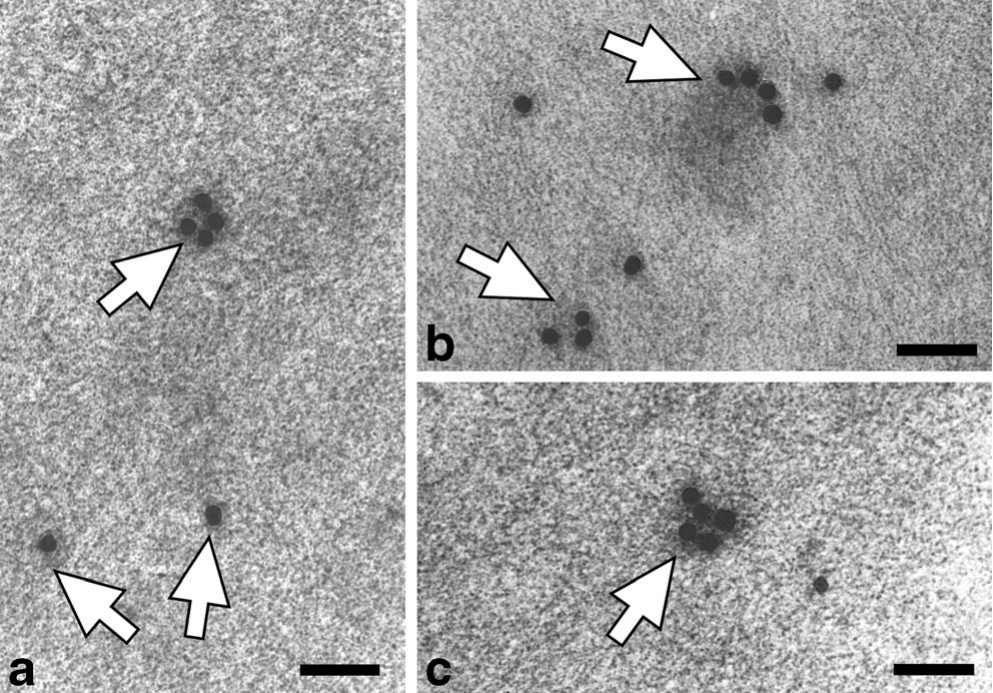
**a**–**c** Marked cutinsomes present in the cell wall (*white arrows*). *Scale bar*, 100 nm

**Fig. 8:**
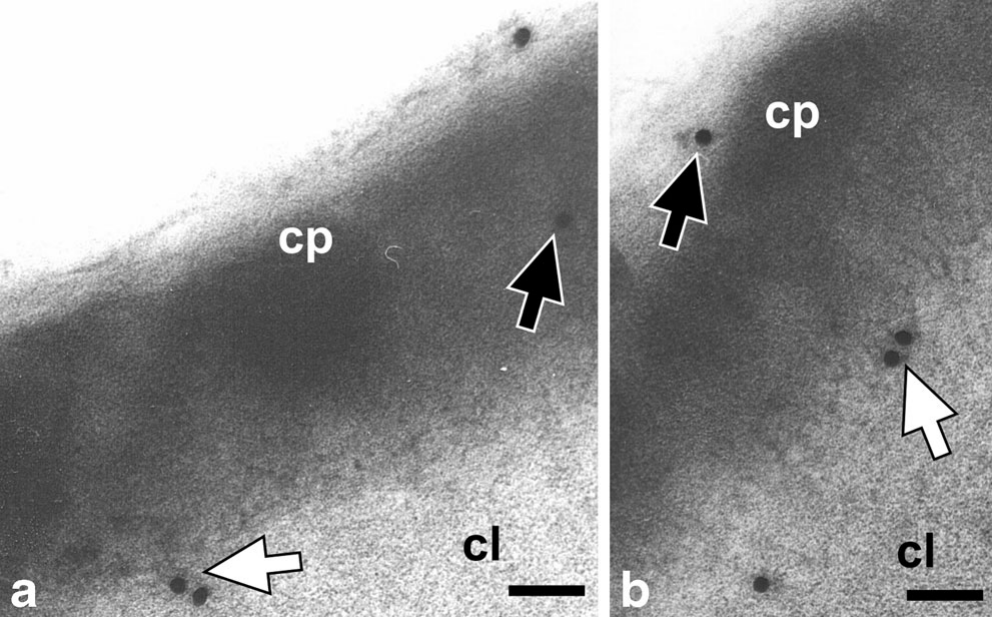
**a**, **b** Labelled cutinsomes in the cuticle. Cutinsomes on the border of the cuticular layer and cuticle proper (*white arrow*) and in the cuticle proper (*black arrow*). *cl* cuticular layer, *cp* cuticle proper. *Scale bar*, 100 nm

**Fig. 9:**
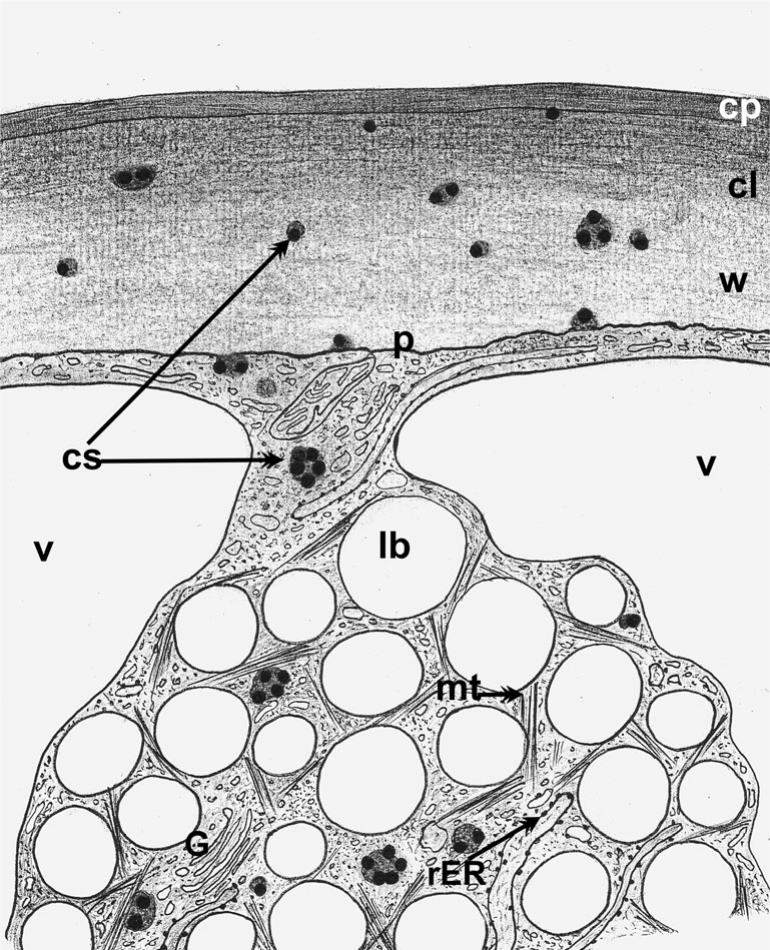
Scheme of cutinsome localization in *O. umbellatum* ovary epidermal cell. The cutinsomes form in a lipotubuloid and then probably move towards the cell wall and cuticle through the plasmalemma (on the basis of enclosed electron microscopy–immunogold technique images). *cl* cuticular layer, *cp* cuticle proper, *cs* cutinsomes, *G* Golgi apparatus, *lb* lipid bodies, *mt* microtubules, *p* plasmalemma, *rER* rough endoplasmic reticulum, *v* vacuole, *w* cell wall

Figures 3, 4, 5, 6, 7, 8 and 9 present cutinsome-like structures of epidermal cell territories, including lipotubuloids marked with colloidal gold in response to the immunogold technique. Therefore, they may be considered as cutinsomes. In Figs. 3a, 4, 6 and 9, lipid bodies of lipotubuloids are visible as white structures because, due to immunogold technique requirements, its dark osmiophilic content was bleached with hydrogen peroxide (see “Materials and methods”). Thus, the outlines of cutinsomes treated with H_2_O_2_ are less pronounced than those of the non-treated ones (Fig. 2a–c). The cutinsomes 40 nm in diameter labelled with two gold particles are present near lipid bodies entwined with microtubules (Fig. 4a). Furthermore, cutinsomes of similar sizes can be seen near a rough ER vesicle (Fig. 4d). Bigger cutinsomes located among lipid bodies are labelled with more (three to four) gold particles, which are placed on their surface (Fig. 4b, c, e). Small cutinsomes may form near the microtubule band present between lipid bodies, as is seen in Fig. 5a, b. A cutinsome labelled with five gold particles, most probably leaving the lipotubuloid and moving towards the cell wall (Fig. 6a), and cutinsomes adhering to the plasmalemma not forming vesicles on the wall side (Fig. 6b) can be observed. These cutinsomes are probably transported through the plasmalemma by means of non-vesicular lipid transport (Lev 2010; Prinz 2010; Samuels and McFarlane 2012). Cutinsomes of different sizes are present in a polysaccharide cell wall and are labelled with gold particles on their periphery (Fig. 7a–c). Gold particles connected with the cutinsomes at the border of the cuticular layer and cuticle proper as well as in the cuticle proper can be observed in Fig. 8a, b.

The analysis of cutinsome localization in the epidermal cells seems to suggest that cutinsomes are formed in lipotubuloids and gradually move towards the cuticular layer and cuticle proper, as was demonstrated in the scheme based on EM images (Fig. 9).

The presented micrographs seem to suggest that lipotubuloids are likely sites of cutin formation mediated by cutinsomes which are formed at the lipotubuloid territory and then transferred through the plasmalemma and cell wall to the cuticle. This hypothesis seems to be supported by the fact that, in epidermal cells, the labelling density was higher in lipotubuloids than in the cytoplasm and was highest in the cell wall (Table 1).

## Discussion

The presented results obtained from EM observations after the use of the immunogold technique revealing cutinsomes in *O. umbellatum* ovary epidermal cells confirmed earlier hypothesis based on ^3^H-palmitic acid autoradiographic studies that lipids originating from lipotubuloids were the cuticle building substances. Thus, these data supported the idea of Heredia-Guerrero et al. (2008, 2011) that cutinsomes could be involved in cutin formation also in other plants, not only in tomato. Recently, cutinsome-like structures were also identified within the cells and in the walls of the internal epidermis of *Olea europea* seed coat (D’Angeli et al. 2013). Importantly, in these cells, the oil bodies are present analogically to the *O. umbellatum* ovary epidermal cells containing lipotubuloid lipid bodies (Kwiatkowska et al. 2012b). The cutinsomes labelled with gold particles, demonstrated in our work, have different shapes and sizes, similarly to the cutinsomes in tomato (Heredia-Guerrero et al. 2011). Reaction with anti-cutinsome antibodies appeared mostly on their surfaces. This result seems to support the conclusion drawn by Domίnguez et al. (2010) that the cutinsome surface which is a hydrophilic shell being a carboxylate/carboxylic phase (Heredia-Guerrero et al. 2011) is an antigen triggering the immunological reaction.

The cutinsomes were described for the first time at the early stage of cuticle development in tomato fruit (Heredia-Guerrero et al. 2008, 2009). This idea was supported *in planta* by the immunogold method with the use of the antibody recognizing cutinsomes in tomato fruit epidermis 5 days after anthesis (Domίnguez et al. 2010). The same antibody was used to test some cutin-related compounds like fruit cuticular waxes and isolated cutin monomers, but they were not recognized by the antiserum, whilst only cutinsomes gave a pronounced response against the antibodies, even at low concentrations (Domίnguez et al. 2010).

However, the EM–immunogold and CD1 transcript-level studies conducted by Yeats et al. (2012) 15 days post-anthesis showed that cuticle development took place extracellularly and was mediated by acyltransferase CD1 (cutin synthase) which showed the activity during in vitro and in vivo cutin synthesis. This enzyme creates cutin at the site of its deposition (Girard et al. 2012; Yeats et al. 2012). Although it seems that cutinsomes are involved in the early stages of cutin synthesis by means of self-polymerisation process (Heredia-Guerrero et al. 2008), further stages of cuticle development are mediated by cutin synthase (Yeats et al. 2012). Therefore, the course of cutin synthesis seems to depend, among others, on the timing of the development of an organ (Pollard et al. 2008).

In the case of *O. umbellatum* epidermal cells, the situation is different from that in tomato fruit. The presence of cutinsomes was observed by our research team during the very dynamic growth of the ovary, at stages 7–9, when it changed into a fruit and the DNA content underwent endoreduplication (Kwiatkowska et al. 2007).

The present observations seem to prove that the formation of cutinsomes took place intracellularly in lipotubuloids in which lipid body fatty acids, resulting from DGAT2-mediated synthesis, were temporarily stored (Kwiatkowska et al. 2011a, 2012a, b). These lipids were lipolysed by lipase localized on the surface of lipid bodies (Kwiatkowska et al. 2011a, 2012b). The presence of small cutinsomes near the lipid bodies suggests that fatty acids resulting from lipolysis could be directly used to form cutinsomes. The cutinsomes *in statu nascendi* were also visible near bands of microtubules which contributed to lipid synthesis as transmitters of fatty acids and enzymes formed in rough ER to lipid bodies; the latter structures were also accompanied by cutinsomes (Kwiatkowska et al. 2012a). However, the site of hydroxylation of fatty acids, which become cutin monomers ready for polymerisation only after hydroxyl group addition (Benίtez et al. 2007, 2008; Heredia-Guerrero et al. 2008, 2009; Domίnguez et al. 2010, 2011), is not known. Elucidation of this issue will require further research with the use of antibodies against the enzymes responsible for this process.

The results of the aforementioned autoradiographic studies prove that the tagged lipids present in lipotubuloids after post-incubation in the non-radioactive medium translocate to the cuticular layer as insoluble in organic solvent components of the cuticle (Kwiatkowska et al. 2011a). Hence, it can be supposed that cutinsomes appear to leave lipotubuloids; pass through the hydrophilic cytoplasm, plasmalemma and polysaccharide cell wall; and finally reach the cuticle. The cutinsomes which are near the plasmalemma which does not form vesicles adhere to it closely (see Fig. 6b, current paper) as they have liquid-like content, so they may easily pass through the membrane (Heredia-Guerrero et al. 2011). Thus, it seems that their transport through this membrane occurs via non-vesicular contact (Lev 2010; Prinz 2010; Samuels and McFarlane 2012). It is possible that the participation of transporters, LTP or ABC, is necessary. The transport of cutinsomes seems to be facilitated by the hydrophilic carboxylate/carboxylic (−COO/COOH) shell surrounding them (Heredia-Guerrero et al. 2011).

In the ovary of *O. umbellatum*, lipotubuloids were present in epidermal cells only; they were not observed in subepidermal parenchymal cells. Thus, the phenomenon described here which was observed in the epidermis is in agreement with previously obtained results showing the presence of factors involved in cuticle synthesis in aerial epidermis (Kolattukudy 2005; Pollard et al. 2008; Lam et al. 2012; Rautengarten et al. 2012). Similarly, in cross-sections of tomato fruit pericarp, cutinsomes were solely observed in epidermal cells (Domίnguez et al. 2010). No lipotubuloids and no cutinsomes, but only a few gold particles (probably as background after immunogold technique), were observed in the subepidermal parenchyma cells of the *O. umbellatum* ovary. The mesophyll cuticle was an exception as it was located under the stomata (Lü et al. 2012).

Moreover, the obtained results seem to support the role of lipotubuloids in cutin synthesis and transport to the cuticle. The fact that lipotubuloids are able to move in a cell together with the cytoplasm streaming (progressive movement) as well as dynamically, autonomously rotate with a speed sixfold greater than that of cyclosis (Kwiatkowska 1972b; Kwiatkowska et al. 2009), undoubtedly facilitates cutinsome release from lipotubuloids and their subsequent transport to a cell wall. Our latest immunogold studies seemed to show that the lipotubuloid’s ability to rotate autonomously resulted from the fact that kinesin and myosin formed the link between microtubules and actin filaments in lipotubuloids (Kwiatkowska et al. 2013).

Lipotubuloids are characteristic of aerial epidermis, not only in *O. umbellatum*. They were described earlier as “elaioplasts” (Wakker 1888), in *Haemanthus albiflos*, *Althaea rosea*, *Vanilla planifolia* and *Funkia sieboldiana* (Kwiatkowska et al. 2010, 2011b). It seems probable that lipotubuloids are very common at least in 120 plant species in which “elaioplasts” were described in the nineteenth and the twentieth century (Kwiatkowska et al. 2011b). Thus, it is likely that the process of cuticle formation, as was described in the present work, may be common among other species, being the *raison d’être* of lipotubuloids in aerial epidermis. Lipotubuloids, which are complex cytoplasmic domains composed of many cooperating organelles, most probably adapted not only to supply cells with nourishment but to produce cutin as well.

In conclusion, it seems possible that the cutinsomes which are formed in lipotubuloids near lipid bodies, microtubules and ER leave the lipotubuloids and move towards the cuticle in dynamically growing epidermal cells of *O. umbellatum* ovary. Similarly, translocation of intracellularly forming cutinsome-like structures from the cytoplasm of the internal epidermis cell seed coat of *O. europea* towards the external cell wall and cuticle was observed (D’Angeli et al. 2013). Thus, we strongly suggest that cutinsomes and lipotubuloids could take part in the synthesis of cuticle components—cutinsomes. This problem requires further research of cutinsomes not only in plant containing lipotubuloids but also in those without lipotubuloids.
